# Editorial: Smart Mobile Data Collection in the Context of Neuroscience

**DOI:** 10.3389/fnins.2021.698597

**Published:** 2021-05-25

**Authors:** Rüdiger Pryss, Berthold Langguth, Thomas Probst, Winfried Schlee, Myra Spiliopoulou, Manfred Reichert

**Affiliations:** ^1^Institute of Clinical Epidemiology and Biometry, University of Würzburg, Würzburg, Germany; ^2^Department of Psychiatry and Psychotherapy, University of Regensburg, Regensburg, Germany; ^3^Department for Psychotherapy and Biopsychosocial Health, Danube University Krems, Krems an der Donau, Austria; ^4^Institute of Technical and Business Information Systems, Otto-von-Guericke-University Magdeburg, Magdeburg, Germany; ^5^Institute of Databases and Information Systems, Ulm University, Ulm, Germany

**Keywords:** mobile data collection, ecological momentary assessments, reviews, meta-analyses, multi-modal data fusion, experience sampling, digital phenotyping, mobile crowdsensing

The Covid-19 pandemic demonstrates both the potential of mobile technology in medicine and the need to exploit this potential (e.g., Zhang et al., 2021). On the flip side, many challenges have to be addressed, for which still no suitable answer exists. This ranges from technical frameworks to the reliability of research data, which have been collected with the help of mobile technology. It is striking that the number of mobile data collection strategies in healthcare grows on a frequent basis. Therefore, overarching topics and considerations are increasingly needed to keep pace with these trends, particularly through their categorization and evaluation. In this Research Topic, such an attempt was pursued for mobile data collection in the context of neuroscience.

**Aim of this Research Topic**: Digital phenotyping, experience sampling, digital health, and ecological momentary assessments (EMA) are only a few methods and strategies that have been presented at the intersection of mobile technology and healthcare. The need to involve multiple disciplines constitutes another important recognition in the given field. Therefore, efforts are constantly needed to review and categorize presented research works. We started this Research Topic in 2018, as we saw and still see many open questions when using mobile technology in healthcare scenarios, especially in the context of clinical neuroscience. The submitted works addressed interesting and novel aspects, also driven through the insight of the identified submission categories. For example, a category multi-modal data fusion could be identified, which will certainly become increasingly important to foster evidence in the context of mobile technology and the neuroscience domain. Altogether, many initially planned aims have been actually pursued by the submissions, but also other directions were presented.

**Overview**: The works submitted to this topic (including rejected papers) cover many aspects of the pursued topic goals. However, we were able to identify three major categories for the contributions: *Experience Sampling, Multi-modal Data Fusion*, and *Reviews and Meta-Analyses*, particularly showing two objectives of the research field of mobile data collection when being used for neuroscience questions. The collection of ecologically valid data through experience sampling or multi-modal data fusion constitutes the first objective. Note that the latter method enables cross-validation of mobile data with other data sources collected through already established methods to gain better insights on the achieved data validity. The second objective, in turn, is concerned with general insights based on Reviews and Meta-Analyses.

## Experience Sampling

The terms Experience Sampling, Ambulatory Assessment and EMA describe a new research method allowing to systematically collect self-reports of emotions, cognition, and behavior in the real-world settings of the participants. A smartphone, wearable or other mobile signaling device is used to prompt the participant with a short questionnaire asking questions on the current situation. This method can then be used to assess fluctuations of clinical symptoms within and between days with high ecological validity and low recall bias (e.g., Probst, 2017; Pryss, 2019).

In this Research Topic, two studies took advantage of this research method: Stieger and Kuhlmann used an experience sampling app to collect a dataset on dreams and nightmares in 92 participants over a period of 22 days. In their paper, they report a detailed item-analysis on the Nightmare Distress Questionnaire and identified those items that reliably discriminate between bad sleep and nightmare. In a study by Weierstall-Pust et al., tablets were used to assess the Post-Traumatic Stress Syndrome in 463 soldiers in Burundi. Based on this field data, the authors were able to discriminate subgroups of soldiers with distinct symptom profiles.

The authors Wurzer and Hauptmann describe an approach, in which a mobile device was used for the treatment of chronic tinnitus. Using a single-arm study design, an auditory stimulation device was tested on a group of 25 tinnitus patients with a treatment period of 16 weeks.

## Multi-modal Data Fusion

The early contribution of Sariyska et al. on feasibility of linking molecular genetic markers to real-world social network size tracked on smartphones linked genetic data to data about the social behavior of people, as recorded with a smartphone app, and reported on an association between genetic expression and social network size of the study participants. This is an example of combining genetic and mobile data for digital phenotyping, and points also to practical limitations: while mobile data are easily scalable, the acquisition of genetic data is an elaborate task, which requires time and money, and for which volunteer recruitment is less easy. This results in smaller samples and less robust models.

Huckins et al. contributed a study on fusing mobile phone sensing and brain imaging to assess depression in college students, in which they identified associations between the time a user had their smartphone unlocked and functional brain activity in brain regions associated with depression. The authors combined passive smartphone sensing data, EMA and functional brain scans and investigated how smartphone usage can be linked to brain connectivity metrics. Their results, derived in one cohort and verified in a second cohort, demonstrate that multi-modal data fusion can lead to new ways of assessing mental health, but also revealed several challenges for the immediate future. Among them, the curse of dimensionality implies that the extraction of knowledge from the high-dimensional fused data requires very large participant samples and dimensionality reduction approaches.

In toward personalized tinnitus treatment: an exploratory study based on internet crowdsensing, Simoes et al. analyzed data of a self-help platform for tinnitus patients to identify predictors of treatment response. Among other findings, the authors showed that treatment duration is the variable explaining most of the variance concerning treatment outcome. Such findings indicate the potential of internet crowdsensing for generating hypotheses for personalized treatments.

In mental condition monitoring based on multimodality biometry, Kiguchi et al. captured multi-modal data to assess mental distress in the workplace. The authors used devices for activity tracking, sleep monitoring, and logging of interactions with office PCs, and they showed that the tracked data agreed with the perceived mental condition of the volunteers, as recorded in questionnaires. Their results suggest that data tracking at the work place has the potential to inform about mental stress.

In motorized shoes induce robust sensorimotor adaptation in walking, Aucie et al. investigated whether results on locomotor adaptation outside the lab agree with insights won in a controlled laboratory environment. They juxtaposed the locomotory behavior of a control group to that of a group wearing motorized shoes with elaborate gear over their normal shoes, and they found that the two groups exhibited mostly the same adaptation patterns. Their results show the potential of wearable devices in modeling movement and gait under real conditions.

## Reviews and Meta-Analyses

Research on smartphones in neuroscience is strongly increasing. In PubMed.gov, the search terms “smartphone AND neuroscience” revealed 51 publications for 2016, 71 publications for 2017, 97 publications for 2018, 122 publications for 2019, and 170 publications for 2020. Reviews and meta-analyses are necessary to synthesize findings for both researchers and practitioners. The review and meta-analysis presented by Goreis et al. examined the potential of smartphone apps to reduce post-traumatic stress symptoms. While a reduction of post-traumatic stress symptoms was observed in participants using apps (g = 0.55), the effect was not significantly stronger than in participants not using the apps (g = 0.09).

Seifert et al. summarized six challenges (collecting data in real-life environments, real-time measurements, within-person data, passive data, smartphone device, data security, and ethical issues) for a smarter smartphone research in neuroscience and provided valuable ideas to overcome these challenges in the future.

The use of smartphone apps to combine mobile crowdsensing and EMA is the focus of the manuscript of Kraft et al. The authors review their experience gathered in various smartphone app projects (TrackYourTinnitus, TrackYourStress, TrackYourHearing, TrackYourDiabetes, Intersession, KINDEX, TinnitusTipps) and provide recommendations for the developments of platforms that combine mobile crowdsensing and ecological momentary assessments.

## Summary

The articles in this special topic provide an overview about the rapidly growing area of mobile data collection in clinical neuroscience. The presented research indicates their huge potential as well as the manifold and multifaceted areas of application.

## Author Contributions

All authors listed have made a substantial, direct and intellectual contribution to the work, and approved it for publication.

## Conflict of Interest

The authors declare that the research was conducted in the absence of any commercial or financial relationships that could be construed as a potential conflict of interest.

## References

[B1] Probst T. (2017). Does tinnitus depend on time-of-day? An ecological momentary assessment study with the “TrackYourTinnitus” application. Front. Aging Neurosci. 9:253. 10.3389/fnagi.2017.00253 28824415 PMC5539131

[B2] Pryss R. (2019). Prospective crowdsensing versus retrospective ratings of tinnitus variability and tinnitus–stress associations based on the TrackYourTinnitus mobile platform. Int. J. Data Sci. Anal. 8, 327–338. 10.1007/s41060-018-0111-4

[B3] Zhang M. W. B. Chow A. Ho R. C. M. Smith H. E. (2021). An overview of commercially available apps in the initial months of the covid-19 pandemic. Front. Psychiatry 12:557299. 10.3389/fpsyt.2021.557299 33935816 PMC8081980

